# Seed viability testing for research and conservation of epiphytic and terrestrial orchids

**DOI:** 10.1186/s40529-022-00333-0

**Published:** 2022-02-10

**Authors:** Namrata Pradhan, Xuli Fan, Francesco Martini, Huayang Chen, Hong Liu, Jiangyun Gao, Uromi Manage Goodale

**Affiliations:** 1 Guangxi Key Laboratory of Forest Ecology and Conservation, College of Forestry, Guangxi University, Daxuedonglu 100, Nanning, Guangxi 530004 People’s Republic of China; 2 State Key Laboratory of Conservation and Utilization of Subtropical Agro-Bioresources, College of Forestry, Guangxi University, Daxuedonglu 100, Nanning, Guangxi 530004 People’s Republic of China; 3 Seed Conservation Specialist Group, Species Survival Commission, International Union for Conservation of Nature (IUCN), 281196 Gland, Switzerland; 4 Lab of Ecology and Evolutionary Biology, Chenggong Campus, Yunnan University, University Town, Chenggong New District, Kunming, Yunnan 650504 People’s Republic of China; 5 State Key Laboratory of Vegetation and Environmental Change, Institute of Botany, The Chinese Academy of Sciences, Beijing, 100093 People’s Republic of China; 6 International Center for Tropical Botany, Department of Earth and Environment, Florida International University, 11200 SW 8th Street Miami, Florida, 33199 USA; 7 Present Address: Faculty of Forest and Wood Sciences, Department of Forest Ecology, Czech University of Life Sciences Prague, Prague, Czech Republic

**Keywords:** Plant conservation, Seed banking, Seed viability test, Orchid seeds, Epiphytic orchids, Terrestrial orchids, Lifeform, Seed sterilization

## Abstract

**Background:**

Seed viability testing is essential in plant conservation and research. Seed viability testing determines the success of *ex-situ* conservation efforts, such as seed banking but commonly testing protocols of orchids lack consistency and accuracy, therefore, there is a need to select an appropriate and reliable viability test, especially when conducting comparative studies. Here, we evaluated the suitability of three seed viability tests, Evans blue test (EB), Fluorescein diacetate test (FDA) and Tetrazolium test (TTC), with and without sterilization, on seeds of 20 orchid species, which included five epiphytes and fifteen terrestrials, using both fresh seeds and seeds stored at − 18 ºC for 6 to 8 years.

**Results:**

We found that sterilization and lifeform of seeds affected seed viability across all tests but the storage time was not an influential factor. Sterilization negatively affected seed viability under EB and FDA test conditions but increased the detection of viable seeds in the TTC test in both epiphytic and terrestrial species. The EB test, when administered without sterilization provided the highest viability results. Being non-enzymatic unlike TTC and FDA tests, as expected, the EB test was the most reliable with similar results between sterilized and not sterilized seeds for most epiphytic and terrestrial species as well as when compared between groups.

**Conclusions:**

The lifeform of the species and seed sterilization prior to testing are important influential factors in orchid seed viability testing. Since EB test was found to be reliable we recommend the EB test for seed viability assessment in orchids rather than the less reliable but commonly used TTC test, or the FDA test, which require more expensive and sophisticated instrumentation. Since storage time was not an influential factor in orchid seed viability testing, the recommendations of this study can be used for both fresh as well as long-term stored orchid seeds. This is helpful for research and especially for conservation measures such as seed banking. However, due to the species specificity of the bio-physiology of orchids, we call for comprehensive viability test assessment in the hyper diverse orchid family to be extended to a greater number of species to facilitate efficient conservation and research.

**Supplementary Information:**

The online version contains supplementary material available at 10.1186/s40529-022-00333-0.

## Background

Seed viability testing, implemented to assess whether seeds are viable and usable after collection or after being in storage, is an integral part of plant research and conservation. A seed viability test is defined as any technique used to determine whether individual seeds appear to be dead or alive within a sample, which enables the proportion of live seeds in a population to be estimated (Gosling 2003). Although conservation in their natural habitat, in situ, is the best option for safeguarding diminishing species numbers, owing to increased habitat loss, fragmentation and habitat degradation, ex situ conservation efforts, such as seed banking (Pant 2013; Schofield et al. 2018). Seed banking provides a long-term security back up for the species and their genetic diversity. Storage conditions are optimized by reducing temperature and relative humidity to ensure that a significant proportion of seeds remain viable during storage for timely regeneration (Magrini et al. 2019; Pritchard and Nadarajan 2008). A viable seed is considered to have achieved the highest physiological maturity that ensure germinaiton under appropriate conditions. In order to retain the seed viability in storage, it is important to collect matured seeds of orchids, since orchid seed viability is highest at the time of physiological maturity, which gradually declines thereafter (Copeland and McDonald 2001). The mature orchid seeds are also desiccation tolerant due to high levels of abscisic acid, low level of moisture content and abundant storage of lipid and protein deposits, and therefore, are considered to have the maximum storage potential (Yeung 2017). However, with time, all seed collections gradually age and decline in viability (Ellis and Roberts 1980; Popova et al. 2016). Therefore, seed viability testing is used to determine the efficacy of collection health and determine recollection efforts (Dalziell and Tomlinson 2017; Fu et al. 2015; Hay and Whitehouse 2017; Walters 2015). Yet, selection of a fast, appropriate and reliable test, especially for comparative assessment can be a challenge in conservation and research (Hay and Whitehouse 2017).

A given seed may contain both live and dead tissues, and a live seed may or may not be capable of germination. Generally, seed viability tests assess whether a seed is alive, metabolically active, and possess enzymes capable of catalyzing metabolic reactions needed for germination and seedling growth (Copeland and McDonald 2001). Thus, seed viability testing can assess tissue viability as well as viability of the entire seed. It can be argued that the simplest and earliest seed tests relied on whether seeds are filled or not, by immersing seeds in water and assigning the seeds that sink to the bottom, filled or good seeds and separating the seeds that would float as “empty” or non-viable. Other tests assess color, appearance, volumetric weight, density and rate of imbibition to indicate seed viability (França-Neto and Krzyzanowski 2019). However, the results of such tests are inaccurate (França-Neto and Krzyzanowski 2019). Germination tests, which do not measure the same property as the viability tests, evaluate the capability of a seed to develop into normal seedlings and are considered a more direct measure of a seed’s capacity to reproduce, but may underestimate seed viability (Gosling 2003). However, knowledge of suitable conditions required for germination as well as the length of time needed to complete the process preclude germination tests from being used in most circumstances (Gosling 2003).

In species targeted for conservation, it is common that seed dormancy breaking techniques may not be effective in promoting germination due to lack of knowledge of the species or lack of adequate quantities of seeds to experimentally gain such knowledge on seed dormancy. In many species, even when such impediments to germination is overcome, germination maybe very slow, requiring long-term germination monitoring. In the former case, it may not be possible to successfully germinate seeds (Yamazaki and Miyoshi 2006) and in the latter the time required to complete the germination test may exceed the standard test times (Koene et al. 2020). This is true especially for orchids, which can take up to several years to complete germination compared to two to three weeks for agricultural and horticultural crops and eight weeks for woody species (Gosling 2003). In this case, a sample set of seeds can be cut and opened to assess the quality of seed tissues (Gosling 2003). However, for many small seeded species, such as the dust like seeds in the family Orchidaceae, cut tests are not practical, neither are X-ray tests that examine and categorize seeds based on how the internal structure of seeds are represented in an X-radiograph (Gosling 2003).

Among numerous other viability tests, some chemical methods rely on the presence or absence of enzymatic activity connected to the live or dead status of a seed or the differential uptake of a stain by living versus dead tissue, commonly known as enzymatic and non-enzymatic tests, respectively (Copeland and McDonald 2001; Wood et al. 2003). These staining tests are based on the premise that viability is an inherent characteristic of a seed’s potential to germinate and, hence, a resting seed is a potential seedling. This pioneering view of characterizing a seed’s viability was developed by Lakon in 1949 (Lakon 1949) through the establishment of the Tetrazolium test (TTC) for seeds. Within hydrated living tissue that contain respiratory enzymes or dehydrogenases, the 2,3,5-triphenyl tetrazolium chloride change from its oxidized, colorless form to the reduced, red or pink color, 2,3,5-triphenyl formazan. The TTC test is one of the most commonly used seed viability tests to date and has the advantage of being a rapid test (Copeland and McDonald 2001). This is especially important for species that are slow and hard to germinate. However, it can be difficult to interpret, requiring experience to determine the test results correctly and results can be inconsistent even within the same seed lot, or within a species (Custódio et al. 2016; de Macedo et al. 2014; Soares et al. 2014).

The fluorescein diacetate test (FDA), which is also suitable for microscopic seeds (Batty et al. 2001; Dowling and Jusaitis 2012; Pritchard 1985; Vendrame et al. 2007; Wood et al. 2003) relies on detecting the fluorescence produced by the living cells, which convert FDA to fluorescein by intracellular esterase enzymes (Dowling and Jusaitis 2012; Wood et al. 2003). The converted fluorescein, being a polar molecule, has a slower rate of escape as compared to the rate of entry of FDA in a cell that contains an intact plasmalemma, causing its accumulation for a period of time within which the fluorescence emanating from seeds’ can be detected using a UV-blue light (Rotman and Papermaster 1966; Wood et al. 2003). Here, seeds stained over the entire embryo surface are considered viable and those seeds that do not show any staining as dead.

The Evans blue test (EB) is a less commonly used non-enzymatic seed viability test (Hooi et al. 2010; Pouzi et al. 2011). Instead of enzymatic activity within the cells, it relies on membrane integrity, with non-viable seeds detected as having a damaged or a leaky membrane, which allows the Evans blue stain to penetrate the cytosol of the embryo cells. The absence of a blue stain within cells indicates that cell membrane pumps are active and hence that seeds are potentially viable. Therefore, seeds with completely unstained embryos are considered as viable and seeds with embryos stained blue as non-viable. The EB test is fast and is considered more accurate but considerable experience and practice is required to handle the Evans Blue solution. In contrast, the TTC and the FDA test can provide false negatives when enzymatic pathways can be temporarily non-functional or give false positives if the enzymes presence after the cell death still result in a viable score (Baker and Mock 1994; Palta et al. 1978). But comparative studies for these rapid staining tests across species and test conditions relevant for conservation objectives are limited especially for the hyper diverse Orchidaceae family.

The entire Orchidaceae family is listed in the Appendix II of Convention on International Trade in Endangered Species (CITES) (Da Silva et al. 2014; Fay 2018; Pant 2013). It also has the most species listed as threatened in the red data book of International Union for Conservation of Nature and Natural Resources (IUCN) (Swarts and Dixon 2009). The reproductive biology and ecological approaches of orchids are complicated and unique (Tsai et al. 2008), requiring efficient investigations on their conservation tools. Orchid conservation efforts heavily rely on seed banking as a conservation tool. Most of the studies on orchid seeds have only used the TTC test for assessing viability in both epiphytic and terrestrial species (Hu et al. 2013; Lakon 1949; Mercado et al. 2020b). However, which test is best suited for correctly estimating seed viability could vary based on lifeforms. Indeed, terrestrial orchids are said to have higher water retention capacity in seeds and more dependency on fungi to germinate as compared to epiphytes (Neto and Custódio 2005; Yoder et al. 2000). The testa of epiphytes is said to be more permeable as compared to terrestrial orchids (Kauth et al. 2008). The seed size also varies from 0.05 to 6.0 mm in length in orchids, with the difference in the shortest and longest seeds being 120-fold (Arditti and Ghani 2000). The differences in these seed traits could also affect stain based rapid test results that assess the physiological condition of seeds. Batty et al. (2001), showed that, all three histochemical staining procedures (TTC, FDA, and EB tests) substantially overestimated seed viability relative to symbiotic germination in four Western Australian terrestrial orchid species. Although in combination, for all four species FDA test was the best predictor of actual germination, they found that there was high unexplained variability among species as well as between treatments. Thus, further investigations are still needed for the comparative assessment of viability outcomes of additional taxa in Orchidaceae representing different lifeforms (Batty et al. 2001).

In this study, we assessed the effect of the viability test (EB, FDA and TTC tests), sterilization prior to testing, species’ life form, i.e., epiphytic or terrestrial, and storage status on orchid seed viability using the seeds of 20 orchid species comprising of five epiphytes and 15 terrestrials (Table 1). We conducted three levels of analyses: for all 20 species using all the above explanatory variables, separately for each lifeform using viability test, sterilization status, storage status, and for each individual species to determine the effect of test type and sterilization. We expected to find lifeform to be an influential factor in determining seed viability of orchid species owing to the variations in the permeability of the testa of epiphytic and terrestrial orchid seeds (Barsberg et al. 2013; Kauth et al. 2008), thus, influencing the permeation of the chemical stains during all biochemical viability testing. Since epiphytic orchids commonly have dry cracks in the testa and terrestrial orchids usually have impermeable testa (Kauth et al. 2008), we expected terrestrial orchids to exhibit lower viability. We expected this outcome to be true especially with TTC and FDA tests, as these tests detect viability with development of stain in the embryo, as compared to epiphytic orchids. We predicted that the sterilization of the seeds prior to the viability tests affects the epiphytic and terrestrial orchids negatively owing to the fact that orchid seeds are dust seeds (Eriksson and Kainulainen 2011), with small size, thin seed coat (Barsberg et al. 2013) and no endosperm (Yeung 2017), making it easy for the sterilizing agents not only to clear the surface contamination but also to penetrate and negatively affect the embryo, thus reducing seed viability. We expected storage time to influence seed viability testing of the seeds with long-term stored seeds to have reduced viability as compared to fresh seeds due to longer time duration from seed collection to viability testing. We expected EB test to be a more stable and reliable test for both epiphytic and terrestrial orchids because EB test is a non-enzymatic test, unlike TTC and FDA tests, and the non-dependency on the enzymes indicates that the EB test will give an unbiased and direct indication of cell death as some of the enzymes may also persist in the seeds after the cell death giving false positive viability results.

**Table 1:** The information of the 20 orchid species used in this study

Sl No.	Species name	Lifeform	Red List Category	Fruit collection site	Fruit collection date
1	*Cymbidium mannii* H. G. Reichenbach	E	NT	XTBG	02 April 2014
2	*Dendrobium cucullatum* R. Brown	E	VU	XTBG	05 March 2013
3	*Acampe joiceyana* (J.J.Sm.) Seidenf	E	NA	XTBG	20 March 2012
4	*Cymbidium floribundum* Lindley	E	VU	YONNR	21 January 2020
5	*Vanda coerulea* Griffith ex Lindley	E	EN	XTBG	16 March 2013
6	*Crepidium purpureum (Lindl.) Szlach*	T	LC	YONNR	21 January 2020
7	*Cymbidium lancifolium* Hooker	T	LC	YONNR	21 January 2020
8	*Cymbidium qiubeiense* K. M. Feng & H. Li	T	EN	YONNR	25 December 2019
9	*Arundina graminifolia* (D. Don) Hochreutiner	T	LC	XTBG	07 February 2012
10	*Liparis nervosa* (Thunberg) Lindley	T	LC	YONNR	25 December 2019
11	*Eulophia zollingeri* (H. G. Reichenbach) J. J. Smith	T	LC	YONNR	26 November 2019
12	*Acanthephippium sylhetense* Lindley	T	VU	XTBG	02 February 2012
13	*Paphiopedilum dianthum* Tang & F. T. Wang	T	*EN, VU	YONNR	21 January 2020
14	*Cymbidium cyperifolium* Wallich ex Lindley	T	VU	YONNR	21 January 2020
15	*Bletilla striata *(Thunberg) H. G. Reichenbach	T	EN	YONNR	26 November 2019
16	*Phaius tancarvilleae* (Banks) Blume	T	LC	XTBG	20 July 2012
17	*Paphiopedilum hirsutissimum* (Lindley ex Hooker) Stein	T	*VU	YONNR	21 January 2020
18	*Liparis nigra* Seidenfaden	T	LC	YONNR	21 January 2020
19	*Cymbidium sinense* (Jackson ex Andrews) Willdenow	T	VU	XTBG	20 January 2012
20	*Calanthe argenteostriata* C. Z. Tang & S. J. Cheng	T	LC	YONNR	21 January 2020

The lifeform, conservation status, fruit collection site, and fruit collection date information of the 20 orchid species used in this study. Lifeform, i.e., epiphyte (E) or terrestrial (T), was assigned based on Flora of China (eFloras, 2020). Conservation status, i.e., Endangered (EN), Vulnerable (VU), Near Threatened (NT), Least Concern (LC), Not Assessed (NA), was assigned based on Chinese red list category (Qin et al*.*, 2017) and IUCN red list category* (IUCN, 2020). The fruit collection sites are Yachang Orchid National Nature Reserve (YONNR) located in Guangxi and Xishuangbanna tropical botanical garden (XTBG) located in Yunnan, China

## Methods

### Study species, seed collection and dehydration

We investigated the reliability of three seed viability tests performed with and without seed sterilization on five epiphytic and 15 terrestrial orchid species collected in Guangxi and Yunnan, Southern China, between 2012 to 2020 (Table 1). A minimum of five individual fully matured fruits; at the verge of dehiscence, were sampled per species and the seeds of the five individuals were mixed together for the experiments. Terrestrial orchids are defined here as species growing on the ground, and includes one saprophytic species as it also grows on the ground. The fruits were collected and transported in labeled paper envelopes and were opened in a sterilized environment in the laminar flow cabinet in respective laboratories in Guangxi and Yunnan. Seeds were dried for five days at room temperature and < 10% humidity using vacuum dryers filled with anhydrous calcium chloride (Hay and Probert 2013) and placed in sealed vials for long-term or short-term storage at − 18 °C, with blue silica gel used as an indicator of relative humidity, from the time of collection until the experimentation. The seeds used in this study were subjected to two storage durations fresh seeds, which were tested for viability within 30 days from the collection date and seeds that were stored for six to eight years. All viability tests and subsequent evaluation of test results were conducted in the Regeneration Ecology, Seed Bio-physiology and Conservation Laboratory at the Forestry College in Guangxi University, Nanning, China.

### Seed viability tests

The methodology for each seed viability test consists of four phases, i.e., sterilization, pre-moistening, incubation and evaluation. Each viability test was conducted in the laminar flow cabinet (Airtech, SW-CJ-2FD, Antai Airtech Company Ltd., Suzhou, China) under a sterile environment, and four replicate seed lots were used for each species and treatment combination. To assess the effect of sterilization on viability testing all three viability tests were also conducted using the same seed lots in replicates of four, following the same procedures but without sterilizing the seeds.

#### Evans blue test (EB test)

**Fig. 1:**
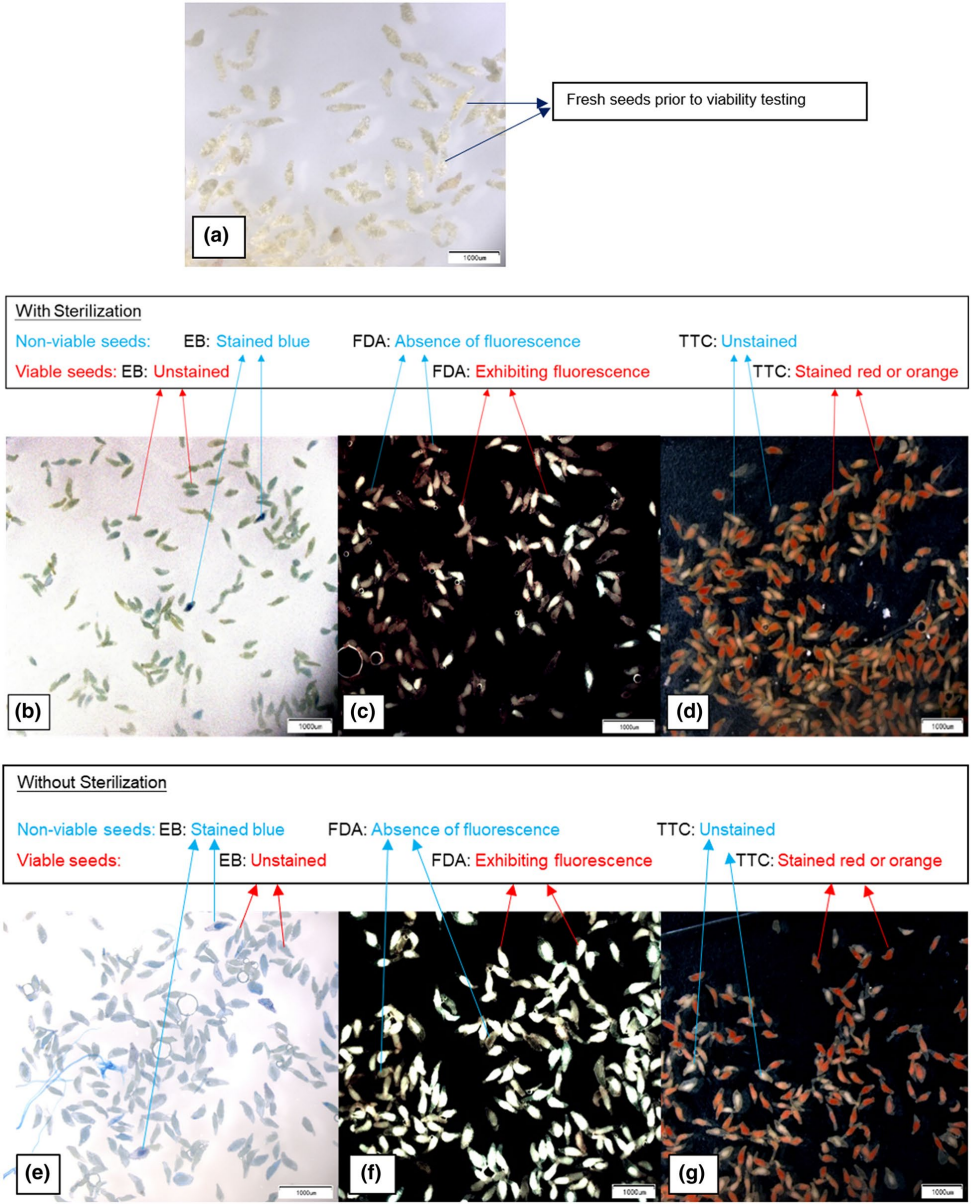
**A–G** Images of seeds of *Cymbidium floribundum*, one of the study species subjected to viability testing. **A** Fresh seeds prior to experimentation. **B–D** Results of viability tests; Evans blue test (EB), Fluorescein diacetate test (FDA) and Tetrazolium test (TTC) performed respectively, after sterilizing the seeds. **E**–**G** EB, FDA and TTC test performed respectively, without seed sterilization

Seed viability was assessed using a modified version of Evans blue test (Batty et al. 2001). The seeds to be tested were sterilized by soaking in 4% (w/v) Ca(OCl)_2_ with 1% (v/v) Tween 80 for 10 min and then, washing the seeds five times thoroughly with distilled water using a syringe fitted with 45 μm mesh. Then, seeds were soaked for 16 h in distilled water, following which the water was discarded and the seeds were suspended for one hour in 1% (w/v) Evans blue solution. The syringe was shaken frequently to facilitate proper contact between the seeds and the Evans blue solution. Finally, seeds were rinsed thoroughly five times with distilled water to remove excess stain and the seeds were placed on a microscopic glass slide, and viewed under the stereomicroscope (Olympus SXZ12, Japan). The seeds with unstained embryos were considered viable and those with embryos stained blue as non-viable (Fig. 1).

#### Fluorescein diacetate test (FDA test)

For the FDA test, modified from Batty et al. (2001), the 0.5% (w/v) solution of fluorescein diacetate (FDA) was prepared by dissolving 3,6-diacetoxyfluoran di-*O*-acetylfluorescein powder in acetone. To conduct the test, orchid seeds were soaked for 15 min in 4% (w/v) Ca(OCl)_2_ and 1% (v/v) Tween 80 after which seeds were rinsed five times thoroughly in distilled water using a syringe fitted with 45 μm mesh and were soaked in distilled water for 16 h. After soaking, they were suspended in FDA solution for 15 min to allow fluorescein to accumulate in living cells. The syringe was shaken frequently to facilitate proper contact between the seeds and the FDA solution. Seeds were then washed once with distilled water to remove excess stain and placed on a microscopic glass slide and examined under the UV-fluorescence microscope (excitation 460–490 nm; Olympus SXZ12, Japan). The seeds with embryos exhibiting fluorescence were considered viable and those with absence of fluorescence as non-viable (Fig. 1).

#### Tetrazolium test (TTC test)

The seed viability with Tetrazolium test was assessed using the modified version of the seed viability method used by Terry et al. (2003) and others (Alvarez-Pardo et al. 2006; Batty et al. 2001; Miller 2010). Seeds were first soaked in 4% (w/v) Ca(OCl)_2_ and 1% (v/v) Tween 80 for 20 min, then washed five times thoroughly with distilled water using a syringe fitted with a 45 μm mesh and suspended in distilled water for 24 h. Then, the water was discarded and 1% (w/v) 2,3,5-triphenyl tetrazolium chloride (TTC) solution was extracted into the syringe containing seeds, and the reaction in the syringe was allowed to take place for 24 h at 30 °C in the dark. The syringe was shaken frequently to facilitate proper contact between the seeds and the TTC solution. After discarding the TTC solution, seeds were washed once with distilled water to remove excess stain and examined under the stereomicroscope (Olympus SXZ12, Japan). Seeds were scored as viable if the embryo was stained red or orange and as non-viable if the embryo was unstained (Fig. 1).

### Assessment and interpretation of viability test results

A minimum of 100 seeds for each replicate and treatment condition was used to assess the viability. The viable and non-viable seeds were counted using a digital image of 10× to 30× magnification captured under the stereomicroscope. The captured images were transferred to Image Processing and Analysis in Java (ImageJ; Schneider et al. 2012) for analysis and scoring (Hay and Whitehouse 2017). All images were scored and assessed by the same individual (NP) to reduce potential biases and inacuracies in the interpretation of the viability results.

### Statistical analyses

All analyses were conducted using R (version 4.0.2; R.Core.Team 2020). The binary response variables were used to predict the probability of finding viable seeds (PSV) from 0 to 1, where 0 is non-viable and 1 is viable by combining the two viable and non-viable responses using the *cbind* function in R. We assessed the best-fit model among the Binomial model, Observation-Level Random Effects model (*lme4* package; Bates et al. 2015) and the Beta-Binomial model (*glmmTMB* package; Brooks et al. 2017) using the Akaike’s information criterion (ΔAIC) values. Overdispersion was checked with the *DHARMa* package (Hartig 2020). The Beta-Binomial model, which employs a Template Model Builder (Brooks et al. 2017) to account for overdispersed binomial data, a common characteristic of biological data models (Harrison 2015), was the best fit to our data. Final models were developed after checking for collinearity among predictors (variance inflation factor < 3) using the ‘vif’ function in the *car* package (Fox and Weisberg 2019). Diagnostic plots were used to check and correct for outliers, influential observations, non-normality, non-constant error variance, multi-collinearity and non-linearity. The figures were developed using *ggplot* (Wickham 2016).

In the first analysis, we explored which factors affected seed viability test results by assessing both the epiphytic and terrestrial orchids together. Our model had seed viability test, sterilization status, the interaction between seed viability test and sterilization status, lifeform and storage status as fixed factors and species as a random factor. The full model had the following form:

1 $$Y \sim { \beta }_{0}+{\beta }_{1}VT+ {\beta }_{2}SZ+{\beta }_{3}LF+{\beta }_{4}SS+{\beta }_{5}V{T}^{ *} {\beta }_{6}SZ+{\varepsilon }_{sp}+{\varepsilon }_{residual}$$

where, *β*_*1*_ to *β*_*6*_ are the parameters to be estimated, *Y* is the PSV, *VT* is viability test type (TTC, FDA, and EB test), *SZ* is sterilization status, *LF* is lifeform and *SS* is storage status. The random effect of species is denoted by *ε*_sp_ and *ε*_residual_ is the residual error. Model averaging was performed on the full model using the *MuMIn* package (Barton 2020) and the results showed that all factors but SS had a clear effect on the response variable based on the 95% confidence intervals not including zero. The results were based on the model-averaged full coefficient.

Next, the analyses were conducted separately for each lifeform, epiphytic and terrestrial (Table 2) to assess how *VT*, *SZ*, the interaction between *VT* and *SZ*, and *SS* (all as fixed factors) affects the PSV for each lifeform (*Y*_*epiphyte*_ or *Y*_*terrestrial*_) in the two lifeforms separately, using the following model:

**Table 2:** The results of response of PSV with three viability tests of epiphytic and terrestrial orchids

Response	Predictor	Estimate	Std. Error	CI	Z value	*P*- value
(a) Epiphytic and Terrestrial orchids						
PSV	(Intercept)	2.48	0.29	1.91 – 3.05	8.52	** < 0.001**
	FDA test vs. EB test	−0.78	0.15	−1.08 – -0.48	−5.1	** < 0.001**
	TTC test vs. EB test	−3.02	0.17	−3.34 – -2.70	−18.29	** < 0.001**
	FDA test vs. TTC test	2.24	0.16	1.93 – 2.55	14.2	** < 0.001**
	Sterilized vs. non-sterilized seeds	-0.85	0.15	−1.15 – -0.55	−5.57	** < 0.001**
	Terrestrial vs. epiphyte lifeform	−0.82	0.26	−1.33 – -0.31	−3.17	**0.002**
	Stored vs. fresh seeds	0.08	0.23	−0.36 – 0.53	0.37	0.712
	FDA test*sterilized and not sterilized seeds vs. EB test *sterilized and not sterilized seeds	−0.17	0.21	−0.58 – 0.24	−0.8	0.422
	TTC test*sterilized and not sterilized seeds vs. EB test*sterilized and not sterilized seeds	1.62	0.22	1.20 – 2.04	7.52	** < 0.001**
	FDA test*sterilized and not sterilized seeds vs. TTC test*sterilized and not sterilized seeds	−1.79	0.21	−2.20 – -1.38	−8.49	** < 0.001**
	AIC = 5941.644; Marginal R^2^ = 0.251; Conditional R^2^ = 0.284					
(b) Epiphytic orchids						
PSV	(Intercept)	2.27	0.33	1.62 – 2.92	6.82	** < 0.001**
	FDA test vs. EB test	−1.1	0.32	−1.72 – -0.48	−3.47	**0.001**
	TTC test vs. EB test	−2.13	0.32	-2.77 – -1.49	−6.57	** < 0.001**
	FDA test vs. TTC test	1.03	0.29	0.46 – 1.60	3.57	** < 0.001**
	Sterilized vs. Not sterilized seeds	−1.01	0.31	−1.63 – -0.40	−3.22	**0.001**
	Stored vs. Fresh seeds	0.07	0.27	−0.45 – 0.59	0.26	0.795
	FDA test*sterilized and not sterilized seeds vs. EB test*sterilized and not sterilized seeds	0.47	0.42	−0.37 – 1.30	1.1	0.272
	TTC test*sterilized and not sterilized seeds vs. EB test*sterilized and not sterilized seeds	1.44	0.42	0.62 – 2.27	3.42	**0.001**
	FDA test*sterilized and not sterilized seeds vs. TTC test*sterilized and not sterilized seeds	−0.98	0.4	−1.76 – -0.19	−2.44	**0.015**
	AIC = 1589.915; Marginal R^2^ = 0.123; Conditional R^2^ = 0.132					
(c) Terrestrial orchids						
PSV	(Intercept)	1.74	0.19	1.37 – 2.10	9.26	** < 0.001**
	FDA test vs. EB test	-0.68	0.17	−1.02 – -0.35	−3.99	** < 0.001**
	TTC test vs. EB test	-3.42	0.2	−3.81 – -3.03	−17.15	** < 0.001**
	FDA test vs. TTC test	2.74	0.19	2.36 – 3.12	14.2	** < 0.001**
	Sterilized vs. Not sterilized seeds	-0.8	0.17	−1.14 – -0.46	−4.62	** < 0.001**
	Stored vs. Fresh seeds	0.1	0.29	−0.47 – 0.67	0.34	0.73
	FDA test*sterilized and not sterilized seeds vs. EB test*sterilized and not sterilized seeds	-0.38	0.24	−0.84 – 0.08	−1.61	0.108
	TTC test*sterilized and not sterilized seeds vs. EB test*sterilized and not sterilized seeds	1.75	0.26	1.25 – 2.25	6.84	** < 0.001**
	FDA test*sterilized and not sterilized seeds vs. TTC test*sterilized and not sterilized seeds	-2.13	0.25	−2.62 – -1.64	−8.49	** < 0.001**
	AIC = 4332.92; Marginal R^2^ = 0.279; Conditional R^2^ = 0.319					

The results of the response of probability of finding viable seeds (PSV) with three viability tests; Evans blue test (EB), Fluorescein diacetate test (FDA) and Tetrazolium test (TTC), with and without sterilization, using Beta-Binomial models, of (a) epiphytic and terrestrial orchids, (b) epiphytic orchids and (c) terrestrial orchids. The bold font values are significant at P < 0.05

2 $${Y}_{epiphyte}\,or\, {Y}_{terrestrial} \sim {\beta }_{0}+{\beta }_{1}VT+ {\beta }_{2}SZ+{\beta }_{3}SS+ {\beta }_{4}V{T}^{ *}{\beta }_{5}SZ {+ \varepsilon }_{sp}+{\varepsilon }_{residual}$$

where, *β*_*1*_ to *β*_*5*_ are the parameters to be estimated, and the same random errors as above were added to this model. Multiple comparisons among the viability tests and their sterilization status for both lifeforms were performed using the Tukey test.

A final species level analysis on each of the 20 orchid species, to determine the effect of *VT* and *SZ* and their interaction, was conducted using the following equation:

3 $${Y}_{species }\sim {\beta }_{0}+{\beta }_{1}VT+ {\beta }_{2}SZ+ {\beta }_{3}V{T}^{ *}{\beta }_{4}SZ+{\varepsilon }_{residual}$$

Here, *Y*_*species*_ is the PSV for each species, *VT*, *SZ* and the interaction of these two variables were set as fixed factors, *β*_*1*_ to *β*_*4*_ are the parameters to be estimated and *ε*_residual_ is the residual error. Within the context of each fitted full model, multicollinearity among fixed factors were tested using the *car* package.

## Results

A total of 480 viability tests were conducted in this study on seeds of 20 orchid species with and without sterilization prior to testing. In the pooled analysis of epiphytic and terrestrial species, the PSV response to each test depended on the test type with estimates varying approximately three-fold higher for the EB test as compared to the TTC test (*P* < 0.001). The PSV also depended on whether the orchid seeds were sterilized or not and whether they were epiphytes or terrestrials, with sterilization lowering the seed viability in the pooled analysis and epiphytes showing greater viability as a group compared to the terrestrial species (*P* = 0.002; Table 2a). Surprisingly, in the species studied here, storage time did not influence the PSV of any viability test in the pooled analysis as well as when each lifeform was analyzed separately (all *P* > 0.7; Table 2a).

**Fig. 2:**
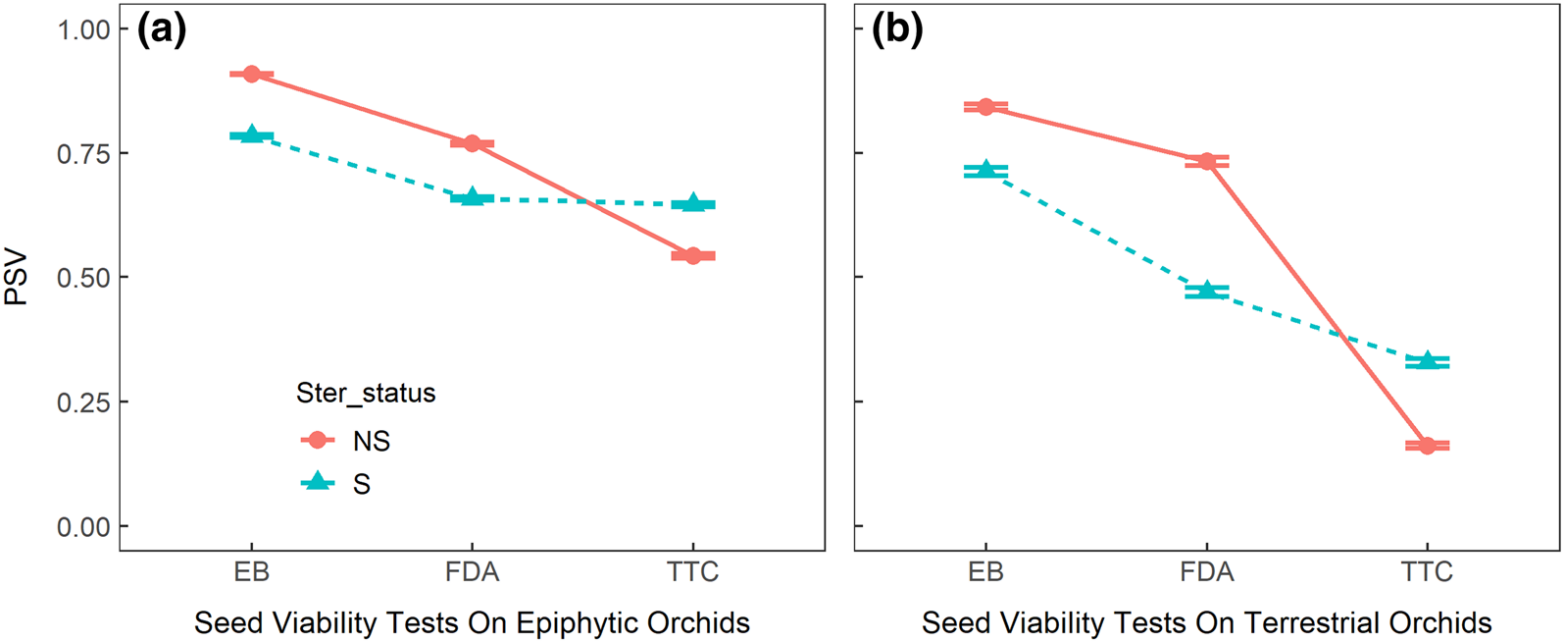
The probability of finding viable seeds (PSV) after subjecting to three seed viability tests; Evans blue test (EB), Fluorescein diacetate test (FDA) and Tetrazolium test (TTC) for (**a**) epiphytic and (**b**) terrestrial orchids, without sterilization (NS) and with sterilization (S) of seeds

**Fig. 3:**
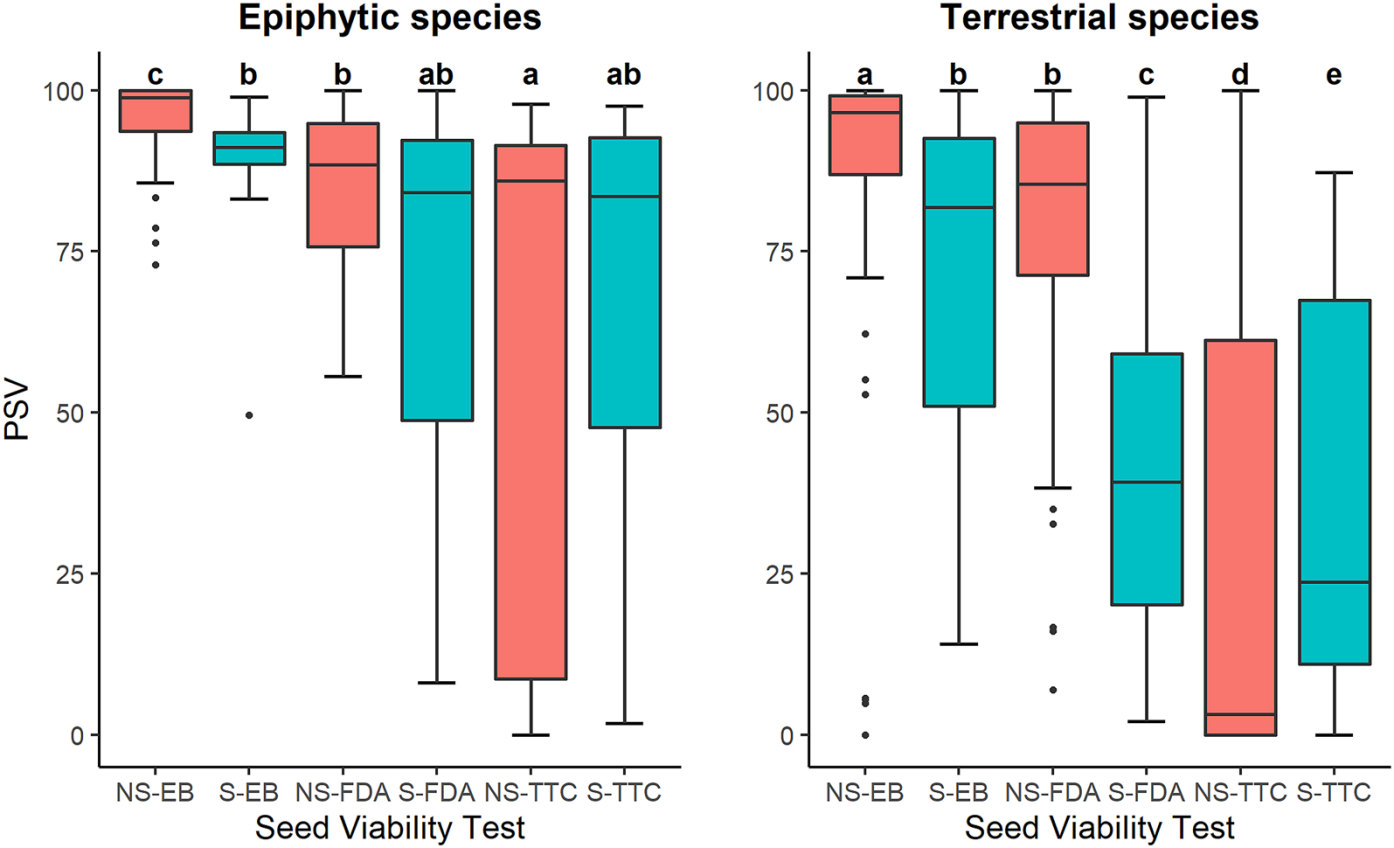
Box plots for the probability of finding viable seeds (PSV) after subjecting to three seed viability tests; Evans blue test (EB), Fluorescein diacetate test (FDA) and Tetrazolium test (TTC) for epiphytic and terrestrial orchids without sterilization (NS) and with sterilization (S) of seeds. Viability tests that are marked with the same letters were similar statistically (*P* > 0.05) while those with different letters were not

A closer look at each lifeform showed that the PSV patterns observed in the pooled analysis remained the same (Fig. 2; Table 2b, c). Sterilization significantly reduced the viability detection probability in the EB and FDA tests but the effect was the opposite for the TTC test, with PSV being greater for sterilized seeds compared to the non-sterilized seeds (*P* < 0.001). The sterilization effect was slightly higher for the epiphytic seeds (effect size -1.01 and -0.8 for epiphytic and terrestrial orchids, respectively; Table 2). Terrestrial orchids exhibited lower viability especially with TTC and FDA tests as compared to epiphytic orchids (Fig. 2). For both epiphytic and terrestrial orchids, PSV was highest for the EB test without sterilization (PSV = 0.91 ± 0.008 and 0.84 ± 0.06 for epiphytic and terrestrial species, respectively, Fig. 2). The lowest PSV was observed when the TTC test was administered on non-sterilized terrestrial orchid seeds (PSV = 0.16 ± 0.05, Fig. 2). When the stability of each viability test was assessed using the PSV variance detected between samples for each lifeform, the greatest stability was detected in the EB test results in epiphytic species with lower variance between maximum and minimum viability obtained (27 variance for non-sterilized seeds; Fig. 3). All other viability tests for both lifeforms showed high PSV variability between the maximum and minimum viability obtained (variance > 44.4, Fig. 3).

At the species level, sterilization was influential on PSV in most species studied (*P* < 0.05; Table 3) except for two epiphytic species and six terrestrial species (*P* > 0.05; Table 3). Even at the species level, among the 20 species, 19 species showed highest PSV with EB test, among which 17 species showed highest PSV for EB test without sterilization and 2 species; *Calanthe argenteostriata* and *Liparis nervosa*, for EB test with sterilization (Additional file 1: Fig. S1). The only exception was *Eulophia zollingeri* which gave highest PSV with FDA test without sterilization (Additional file 1: Fig. S1). At the species level, for the TTC test, 12 species showed greater viability with sterilized seeds as compared to without sterilization, unlike EB and FDA tests (Additional file 1: Fig. S1). Among the three viability tests, the EB test gave highest number of species (ten species) with minimum difference of mean PSV when tested with and without sterilization of seeds and lowest number of species (three species) with maximum difference of mean PSV for seeds tested with and without sterilization of seeds (Additional file 1: Table S1).

**Table 3:** Results of Beta-Binomial models tested for 20 orchid species

	*χ2*	*P*	χ2	*P*	χ2	*P*
Seed viability	VT		SZ		VT*SZ	
P_via-1_	480.344	< 2e−16 ***	0.646	0.42156	11.679	0.00291 **
P_via-2_	269.542	< 2.2e−16 ***	77.697	< 2.2e−16 ***	195.119	< 2.2e−16 ***
P_via-3_	127.705	< 2.2e−16 ***	110.001	< 2.2e−16 ***	62.926	2.167e−14 ***
P_via-4_	49.755	1.570e−11 ***	33.953	5.646e−09 ***	12.054	0.002413 **
P_via-5_	5.1986	0.07432	1.0714	0.30063	5.1158	0.07747
P_via-6_	371.7443	< 2.2e−16 ***	0.0154	0.9012717	13.9882	0.0009173 ***
P_via-7_	251.6452	< 2e−16 ***	273.5214	< 2e−16 ***	0.4325	0.8055
P_via-8_	213.2834	< 2.2e−16 ***	4.5755	0.03243 *	23.7845	6.843e−06 ***
P_via-9_	207.9811	< 2e−16 ***	0.0178	0.8937	358.7346	< 2e−16 ***
P_via-10_	198.982	< 2.2e−16 ***	30.329	3.646e−08 ***	61.94	3.548e−14 ***
P_via-11_	175.4492	< 2.2e−16 ***	7.5146	0.00612 **	59.2462	1.364e−13 ***
P_via-12_	102.5499	< 2.2e−16 ***	0.2353	0.6276	41.7082	8.773e−10 ***
P_via-13_	61.927	3.57e−14 ***	77.749	< 2.2e−16 ***	10.303	0.00579 **
P_via-14_	49.9478	1.426e−11 ***	4.8081	0.02833 *	46.3528	8.603e−11 ***
P_via-15_	49.848	1.498e−11 ***	39.416	3.425e−10 ***	15.996	0.0003362 ***
P_via-16_	42.207	6.837e−10 ***	0.1602	0.689	66.6631	3.344e−15 ***
P_via-17_	31.365	1.546e−07 ***	36.794	1.313e−09 ***	19.571	5.626e−05 ***
P_via-18_	21.3451	2.317e−05 ***	2.767	0.09623	0.4886	0.78325
P_via-19_	16.3238	0.0002853 ***	2.399	0.1214103	5.7222	0.0572067
P_via-20_	6.8054	0.033284 *	9.9687	0.001592 **	21.7605	1.883e−05 ***

Results of Beta-Binomial models tested for 20 orchid species. P_via_ is the viability percentage as the response for each species with serial number 1 to 20 as allocated in the Table 1, for all epiphytic species (1–5), followed by all terrestrial species (6–20). The VT is the viability test, SZ is the sterilization status and VT*SZ is the interaction between VT and SZ. The values marked *** are of highest significance (p < 0.001)

## Discussion

Physiological tools, such as a reliable seed viability test, can be used to effectively solve conservation problems (Cooke et al. 2013). In the case of orchid conservation through seed banking, seed viability test is an essential tool to design and manage seed conservation. A greater understanding of the factors that affect the accuracy and reliability of seed viability tests could facilitate determining the most intact physiological status of seeds to be conserved, increasing the success and efficiency of seed banking or research for conservation (Dalziell and Tomlinson 2017). Orchid seeds are ideal resources for seed banking as they are dust-like seeds, minute in size (Yang and Lee 2014) and weight, which enables large volume storage without the need for large facilities (Magrini et al. 2019). This study showed that the three most commonly used seed viability tests for orchid species showed significant variation in PSV among the three test types, EB test, FDA test and TTC test, depending on the species’ lifeform, i.e., whether they were epiphytic or terrestrial and seed sterilization prior to testing. The variation in the PSV of epiphytic and terrestrial orchids with the seed viability tests could be attributed to the differences in the permeability of their testa (Dowling and Jusaitis 2012; Lauzer et al. 2007; Pritchard 1985; Wood et al. 2003); epiphytic orchids commonly have dry cracks in the testa whereas terrestrial orchids usually have impermeable testa (Kauth et al. 2008). The impermeability is due to the presence of suberin, a waxy substance, found on the testa of orchid seeds (Barsberg et al. 2013; Kauth et al. 2008). As a result, as expected, terrestrial orchids exhibited lower viability especially with TTC and FDA tests as compared to epiphytic orchids (Fig. 2). Contrary to our expectation, storage time did not affect the viability results for the species studied here. This indicates that eight years of storage at − 18 °C was not long enough to cause significant degradation of seed viability in our study species.

The TTC and FDA tests are enzymatic tests that rely on specific enzymes; TTC test relies on dehydrogenase enzyme and FDA test relies on esterase enzyme, whereas the EB test is not dependent on any enzymes but rather the membrane integrity (Hooi et al. 2010) where the Evans blue stain acts as a non-permeating dye which can only leak through ruptured membranes and stain the contents of dead cells (Hooi et al. 2010; Keßler and Furusaki 1997; Zainuddin et al. 2011). As a result, EB test has been used in studies as a direct indication of cell death (Baker and Mock 1994; Hooi et al. 2010; Pouzi et al. 2011; Zainuddin et al. 2011). A non-enzymatic test, such as the EB test, is more reliable to test the seed viability as compared to enzymatic tests because some of the enzymes can also persist in the seeds even after the cell death, thus, providing a false positive (Palta et al. 1978). In our results, we saw that the EB test has the highest PSV across all species, except *E. zollingeri*. EB test gave similar results with and without sterilization between epiphytic and terrestrial orchids while the other two tests, FDA and TTC test exhibited more variations between their results with and without sterilization (Fig. 2). The EB test gave highest number of species (ten species) with minimum difference in mean PSV for seeds with and without sterilization and the lowest number (three species) of species with maximum difference of mean PSV for seeds with and without sterilization of seeds (Additional file 1: Table S1). This indicates that EB test is a more reliable test as compared to other two viability tests for terrestrial as well as epiphytic orchids. Therefore, we recommend EB test to be preferred for seed viability testing of epiphytic and terrestrial orchids.

The FDA test has shown variable results with orchids in the past (Batty et al. 2001; Dowling and Jusaitis 2012). Some studies claim FDA test to provide more accurate estimate of viability as compared to TTC test (Dowling and Jusaitis 2012). It has been stated that the pre-sterilization phase before FDA test cause a modest stress to the embryo and lowers the staining ability of the embryo with FDA stain (Wood et al. 2003). This theory was supported in this study too as we found lower PSV when using FDA test with sterilization as compared to without sterilization for four epiphytic and 13 terrestrial species (Additional file 1: Fig. S1).

The TTC test have been used in various orchid studies but the results have been variable and inconsistent even within species (Alvarez-Pardo et al. 2006; Custódio et al. 2016; de Macedo et al. 2014; Dowling and Jusaitis 2012; Hosomi et al. 2011, 2012; Lallana and García 2013; Soares et al. 2014; Vujanovic et al. 2000; Yamazaki and Miyoshi 2006). The instability with the TTC test was seen in this study too. It resulted in the lowest PSV for most species, especially when the TTC test was administered without seed sterilization as compared to FDA and EB tests (Additional file 1: Fig. S1). Further, the TTC results with and without sterilization of seeds had maximum variation in eight species (Additional file 1: Table S1), indicating its instability as a viability test.

The main challenge of doing these biochemical seed viability tests on orchids is that it requires personnel with good vision, fine surgical skills, meticulous attention to detail, great care, regular practice, considerable patience, extensive experience and an experienced trainer (Gosling 2003). In our study, for orchid species that have a dark colored testa, such as the seeds of *Paphiopedilum* species that are naturally brown in color, the evaluation of the seed viability with TTC test was more challenging as the natural color of the testa creates confusion with the red stain within the seeds stemming from the TTC solution (Dowling and Jusaitis 2012). Such problems have been reported with other orchid species that have brown embryos like *Epipactis* species (Wood et al. 2003). For such species with dark colored testa or embryo, we recommend testing viability with FDA test as it is a fluorescent based test making it more convenient to estimate seed viability in species with dark colored seeds.

Sterilization was an important factor that affected the seed viability results, where sterilized seeds were more likely to show decreased viability of orchids, especially with the EB and FDA tests. This negative effect of sterilization on orchid seed viability can be attributed to the fact that orchid seeds are dust seeds (Eriksson and Kainulainen 2011), with small size, thin seed coat (Barsberg et al. 2013) and no endosperm (Yeung 2017), which makes it easy for the sterilizing agents to penetrate inside the seeds and affect the embryo (Wood et al. 2003). However, this result was not consistent for the TTC test, where both at the lifeform level and the species level, greater viability was observed with the TTC test for the samples that were subjected to sterilization. This can be credited to the fact that sterilization with hypochlorite solutions (Miyoshi and Mii 1998) before vital staining can breakdown the seed coat resulting in greater permeability, which can then increase the effectiveness of the vital stain (Sawma and Mohler 2002; Van Waes and Debergh 1986). Therefore, in general, care must be taken to find the optimal type and concentration of the sterilizing agent for each orchid species that does not damage the embryo but only provide an effective surface sterilization (Kauth et al. 2008).

Since the storage status; fresh seeds and long-term stored seeds, was found to be insignificant in both lifeforms, this indicates that the recommendations of this study can be applied for viability testing of fresh as well as long-term stored seeds; 6 to 8 years storage. This makes it easy for research and especially for conservation measures such as seed banks, where fresh seeds post collection needs to be investigated correctly for initial seed viability before storage and the long-term stored seeds need to be monitored regularly to see if the seed viability is intact during storage (Dalziell and Tomlinson 2017; Dowling and Jusaitis 2012; Hay and Whitehouse 2017; Seaton et al. 2015). Orchid seed longevity can be enhanced by reducing moisture content and lowering storage temperatures and thus are generally regarded as “orthodox” (Pritchard et al. 1999). However, there are some orchid species that are more sensitive to desiccation and storage than others, showing variable results of deterioration in germinability after the same storage durations, as compared to their germination prior to storage, especially noticed in conventional seed banking (Popova et al. 2016).

In most of the studies involving viability tests and germination tests, there have not been a clear distinction between seed ‘viability’ and seed ‘germinability’, which creates a false impression that a ‘viable seed’ is synonymous to a ‘germinable seed’ (Gosling 2003). A ‘viable seed’ is one that is alive while a ‘germinable seed’ is a seed exhibiting the emergence of embryonic plant consisting of a complete root and shoot axis that has the capacity of normal growth under favorable conditions (Copeland and McDonald 2001). Therefore, a viability test does not measure the same property as the germination test; a viability test is a measure of physiological status of the seed while a germination test measures the proportion of seeds that are specifically capable of germinating into ‘normal seedlings’ (Gosling 2003). However, conducting germination tests to determine to what extent viable seeds are also germinable should be incorporated as an important step in conservation efforts as the final aim is to be able to conserve seeds that can generate a healthy future population.

As with any experimental study, our study results should be considered with several caveats in mind. First, due to time and resources constraint in this study we focused on seed viability and hence do not provide comparative germinability data. Although we identified sterilization as an important factor influencing PSV, we did not determine the optimum sterilization conditions for each species, which is another important step to be determined for each species before their seeds are subjected to long-term storage. Lastly, a more balanced study design with greater number of epiphytic species, although our models could accommodate unequal sample size, would have provided more clarity to our results. Nonetheless, as far as we know, this is the first study that assessed the most commonly used seed viability test methods, with and without sterilization, to compare outcomes in two orchid lifeforms; epiphyte and terrestrial. Among the 20 species, just five species had been investigated in the past for their seed viability, all of which was completed using just one test, the TTC test. Therefore, further research on seed viability testing on orchids of different lifeforms will improve our efficiency of orchid seed conservation and research.

Seed viability testing is the fundamental step in any kind of plant research as well as their conservation such as seed banking. Therefore, accurate assessment of seed viability is needed (Dalziell and Tomlinson 2017) due to which selection of the most unbiased and reliable viability test is necessary (Hosomi et al. 2017; Mercado et al. 2020a). We urge that the EB test be recognized as a more reliable test for orchid seed viability testing as compared to TTC test and to include the EB test, in the international seed testing rules.

## Conclusions

This study concludes that lifeform of the species and seed sterilization prior to testing are influential factors for orchid seed viability and that EB test is a more reliable seed viability test as compared to FDA and TTC tests. Therefore, this study recommends EB test for seed viability testing of epiphytic and terrestrial orchids. Since there was no significant difference in the storage status; fresh and long-term stored seeds, for both epiphytic and terrestrial orchids, the recommendations of this study are useful for testing both fresh as well as long-term stored orchid seeds, making it especially useful for seed banks. Knowing the viability test that gives reliable seed viability estimate helps minimize errors in the initial steps of the research, therefore, speeds up the research process, and improves efficiency of the conservation measures of species such as seed banking. Thus, this study provides an improvement to the research and conservation of orchids.

## Supplementary Information

**Additional file 1: Table S1.** The mean probability of finding viable seeds (PSV) without sterilization (NS) and with sterilization (S) of seeds, and the difference between NS and S for Evans blue test (EB), Fluorescein diacetate test (FDA) and Tetrazolium test (TTC) for each of the 20 orchid species. The viability test exhibiting maximum and minimum difference between NS and S for each species. The number of species having maximum and minimum difference for each viability test is summarized at the end. **Figure S1.** Box plot for the probability of finding viable seeds (PSV) after subjecting seeds of five epiphytic (first row) and 15 terrestrial orchid species (second to last row) to three seed viability tests; Evans blue test (EB), Fluorescein diacetate test (FDA) and Tetrazolium test (TTC) without sterilization (NS) and with sterilization (S) of seeds. Viability tests marked with the same letters were similar statistically (*P* > 0.05) while those with different letters were not.

## Data Availability

Data are available at Figshare Digital Repository: 10.6084/m9.figshare.19195628.

## Abbreviations

EB: Evans blue test
FDA: Fluorescein diacetate test
TTC: Tetrazolium test
CITES: Convention on International Trade in Endangered Species
IUCN: International Union for Conservation of Nature and Natural Resources
Ca(OCl)_2_: Calcium hypochlorite
ImageJ: Image Processing and Analysis in Java
PSV: Probability of finding viable seeds
AIC: Akaike’s information criterion
VT: Viability test type (TTC, FDA, and EB test)
SZ: Sterilization status
LF: Lifeform
SS: Storage status
