# Correction to: Seed viability testing for research and conservation of epiphytic and terrestrial orchids

**DOI:** 10.1186/s40529-022-00340-1

**Published:** 2022-04-01

**Authors:** Namrata Pradhan, Xuli Fan, Francesco Martini, Huayang Chen, Hong Liu, Jiangyun Gao, Uromi Manage Goodale

**Affiliations:** 1 Guangxi Key Laboratory of Forest Ecology and Conservation, College of Forestry, Guangxi University, Daxuedonglu 100, Nanning, Guangxi 530004 People’s Republic of China; 2 State Key Laboratory of Conservation and Utilization of Subtropical Agro-Bioresources, College of Forestry, Guangxi University, Daxuedonglu 100, Nanning, Guangxi 530004 People’s Republic of China; 3 Seed Conservation Specialist Group, Species Survival Commission, International Union for Conservation of Nature (IUCN), 281196 Gland, Switzerland; 4 Lab of Ecology and Evolutionary Biology, Chenggong Campus, Yunnan University, University Town, Chenggong New District, Kunming, Yunnan 650504 People’s Republic of China; 5 State Key Laboratory of Vegetation and Environmental Change, Institute of Botany, The Chinese Academy of Sciences, Beijing, 100093 People’s Republic of China; 6 International Center for Tropical Botany, Department of Earth and Environment, Florida International University, 11200 SW 8th Street Miami, Florida, 33199 USA; 7 Present Address: Faculty of Forest and Wood Sciences, Department of Forest Ecology, Czech University of Life Sciences Prague, Prague, Czech Republic

## Correction to: Botanical Studies (2022) 63:3 https://doi.org/10.1186/s40529-022-00333-0

In the publication of this article (Pradhan et al. 2022), there was an error in the current (3rd) affiliation for Francesco Martini. The error: “^7^Present Address: Center for Interdisciplinary Research On Ecology and Sustainability, National Dong Hwa University, Hualien 97401, Hualien, Taiwan.” Instead his address is: “^7^Present Address: Faculty of Forest and Wood Sciences, Department of Forest Ecology, Czech University of Life Sciences Prague, Prague, Czech Republic.”

The publication did not contain the following information about the data availability and the ORCID ids of the six authors:

### Availability of data and materials

Data are available at Figshare Digital Repository: https://doi.org/10.6084/m9.figshare.19195628.

The ORCID ids for the following authors are:

- Namrata Pradhan: 0000-0001-9171-8867
- Xuli Fan: 0000-0002-8194-3064
- Francesco Martini: 0000-0003-0806-3930
- Huayang Chen: 0000-0003-4689-812X
- Hong Liu: 0000-0002-7814-5512
- Jiangyun Gao: 0000-0003-1541-5922

The original article (Pradhan et al. 2022) has been corrected.
