# Supplementary material for: Seed viability testing for research and conservation of epiphytic and terrestrial orchids
Source: Bot Stud. 2022 Feb 10;63:3. doi: 10.1186/s40529-022-00333-0 (PMC8831675; doi:10.1186/s40529-022-00333-0)
Supplement: Supplementary file 1 — Additional file 1: Table S1. The mean probability of finding viable seeds (PSV) without sterilization (NS) and with sterilization (S) of seeds, and the difference between NS and S for Evans blue test (EB), Fluorescein diacetate test (FDA) and Tetrazolium test (TTC) for each of the 20 orchid species. The viability test exhibiting maximum and minimum difference between NS and S for each species. The number of species having maximum and minimum difference for each viability test is summarized at the end. Figure S1. Box plot for the probability of finding viable seeds (PSV) after subjecting seeds of five epiphytic (first row) and 15 terrestrial orchid species (second to last row) to three seed viability tests; Evans blue test (EB), Fluorescein diacetate test (FDA) and Tetrazolium test (TTC) without sterilization (NS) and with sterilization (S) of seeds. Viability tests marked with the same letters were similar statistically (P > 0.05) while those with different letters were not. [file 40529_2022_333_MOESM1_ESM.docx]

Seed viability testing for research and conservation of epiphytic and terrestrial orchids

Additional material

Namrata Pradhan^1,2,3#^, Fan Xuli^4#^, Francesco Martini^1,2^, Huayang Chen^5^, Hong Liu^6^, Jiangyun Gao^4^ , Uromi Manage Goodale^1,2,3*^

^1^Guangxi Key Laboratory of Forest Ecology and Conservation, College of Forestry, Guangxi University, Daxuedonglu 100, Nanning, Guangxi 530004, P.R. China

^2^ State Key Laboratory of Conservation and Utilization of Subtropical Agro-bioresources, College of Forestry, Guangxi University, Daxuedonglu 100, Nanning, Guangxi 530004, P.R. China

^3^ Seed Conservation Specialist Group, Species Survival Commission, International Union for Conservation of Nature (IUCN), Gland 281196, Switzerland

^4^Lab of Ecology and Evolutionary Biology, Chenggong Campus, Yunnan University, University Town, Chenggong New District, Kunming, Yunnan 650504, P.R. China

^5^State Key Laboratory of Vegetation and Environmental Change, Institute of Botany, The Chinese Academy of Sciences, Beijing 100093, P.R. China

^6^International Center for Tropical Botany, Department of Earth and Environment, AHC5-387, Florida International University, 11200 SW 8^th^ Street Miami, Florida 33199 USA

Email addresses: NP: [namrata.pradhan@outlook.com](mailto:namrata.pradhan@outlook.com); FX: [38247272@qq.com](mailto:38247272@qq.com); FM: [franmart12@hotmail.it](mailto:franmart12@hotmail.it); HC: [hychen.gxu@outlook.com](mailto:hychen.gxu@outlook.com); HL: [hliu@fiu.edu](mailto:hliu@fiu.edu); JG: [jiangyun.gao@ynu.edu.cn](mailto:jiangyun.gao@ynu.edu.cn); UMG: [uromi.manage.goodale@outlook.com](mailto:uromi.manage.goodale@outlook.com); [uromi.goodale@aya.yale.edu](mailto:uromi.goodale@aya.yale.edu)

^#^The authors contributed equally.

* Author for correspondence: UMG: [uromi.manage.goodale@outlook.com](mailto:uromi.manage.goodale@outlook.com); [uromi.goodale@aya.yale.edu](mailto:uromi.goodale@aya.yale.edu)

Phone: +86-18174128535

**Table S1.** The mean PSV and the difference between NS and S for the 20 orchid species.

| Sl No. | Species | The mean probability of finding viable seeds (PSV) | | | | | | | | | | |
| --- | --- | --- | --- | --- | --- | --- | --- | --- | --- | --- | --- | --- |
|  |  | NS EB | S EB | Difference in EB | NS FDA | S FDA | Difference in FDA | NS TTC | S TTC | Difference in TTC | Maximum difference test | Minimum  difference test |
| 1 | *Cymbidium mannii* | 100 | 92.9 | 7.1 | 97.8 | 93.5 | 4.3 | 0.74 | 3.34 | 2.6 | EB | TTC |
| 2 | *Dendrobium cucullatum* | 99.8 | 94.2 | 5.6 | 95.3 | 94.1 | 1.2 | 2.5 | 81.2 | 78.7 | TTC | FDA |
| 3 | *Acampe joiceyana* | 99.7 | 88.3 | 11.4 | 71.6 | 89.4 | 17.8 | 15.7 | 83.6 | 67.9 | TTC | EB |
| 4 | *Cymbidium floribundum* | 89.8 | 85.8 | 4 | 81.1 | 42.4 | 38.7 | 85.6 | 74.8 | 10.8 | FDA | EB |
| 5 | *Vanda coerulea* | 97.8 | 89.2 | 8.6 | 86.8 | 88.3 | 1.5 | 93.9 | 93.7 | 0.2 | EB | TTC |
| 6 | *Crepidium purpureum* | 98.3 | 97.6 | 0.7 | 96.7 | 92.3 | 4.4 | 2.35 | 9.98 | 7.63 | TTC | EB |
| 7 | *Cymbidium lancifolium* | 99.7 | 83 | 16.7 | 94.5 | 10.7 | 83.8 | 0.12 | 55.9 | 55.78 | FDA | EB |
| 8 | *Cymbidium qiubeiense* | 87.5 | 87.2 | 0.3 | 75.5 | 51.8 | 23.7 | 2.5 | 13 | 10.5 | FDA | EB |
| 9 | *Arundina graminifolia* | 100 | 94.4 | 5.6 | 72.2 | 20.7 | 51.5 | 13.2 | 81.6 | 68.4 | TTC | EB |
| 10 | *Liparis nervosa* | 95.9 | 99.5 | 3.6 | 84 | 97.3 | 13.3 | 55.2 | 19.8 | 35.4 | TTC | EB |
| 11 | *Eulophia zollingeri* | 86.6 | 90.6 | 4 | 96.7 | 46.5 | 50.2 | 0.89 | 0.009 | 0.8 | FDA | TTC |
| 12 | *Acanthephippium sylhetense* | 78 | 64.5 | 13.5 | 38.5 | 55.4 | 16.9 | 0.002 | 69.2 | 69.2 | TTC | EB |
| 13 | *Paphiopedilum dianthum* | 88.8 | 49.9 | 38.9 | 82.2 | 34.3 | 47.9 | 42.7 | 23 | 19.7 | FDA | TTC |
| 14 | *Cymbidium cyperifolium* | 93.8 | 79.4 | 14.4 | 79.3 | 31.1 | 48.2 | 3.7 | 40 | 36.3 | FDA | EB |
| 15 | *Bletilla striata* | 90.2 | 86.3 | 3.9 | 76.6 | 18.3 | 58.3 | 82.7 | 59.1 | 23.6 | FDA | EB |
| 16 | *Phaius tancarvilleae* | 97.9 | 86.2 | 11.7 | 65.2 | 59.4 | 5.8 | 0.6 | 76.5 | 75.9 | TTC | FDA |
| 17 | *Paphiopedilum hirsutissimum* | 96 | 49.9 | 46.1 | 87.4 | 17.5 | 69.9 | 41.7 | 23.5 | 18.2 | FDA | TTC |
| 18 | *Liparis nigra* | 94.4 | 80.4 | 14 | 83.1 | 72.5 | 10.6 | 0.5 | 19.6 | 19.1 | TTC | FDA |
| 19 | *Cymbidium sinense* | 96.5 | 55 | 41.5 | 87.6 | 76.5 | 11.1 | 7.3 | 15.9 | 8.6 | EB | TTC |
| 20 | *Calanthe argenteostriata* | 27.2 | 59.8 | 32.6 | 30.2 | 17 | 13.2 | 6 | 49.5 | 43.5 | FDA | TTC |
|  |  |  |  |  |  |  |  |  |  | Total species: | EB= 3  FDA= 9  TTC= 8 | EB= 10  FDA= 3  TTC= 7 |

**Table S1.** The mean probability of finding viable seeds (PSV) without sterilization (NS) and with sterilization (S) of seeds, and the difference between NS and S for Evans blue test (EB), Fluorescein diacetate test (FDA) and Tetrazolium test (TTC) for each of the 20 orchid species. The viability test exhibiting maximum and minimum difference between NS and S for each species. The number of species having maximum and minimum difference for each viability test is summarized at the end.

**Figure S1.** Boxplot of PSV with three seed viability tests of 20 orchid species.


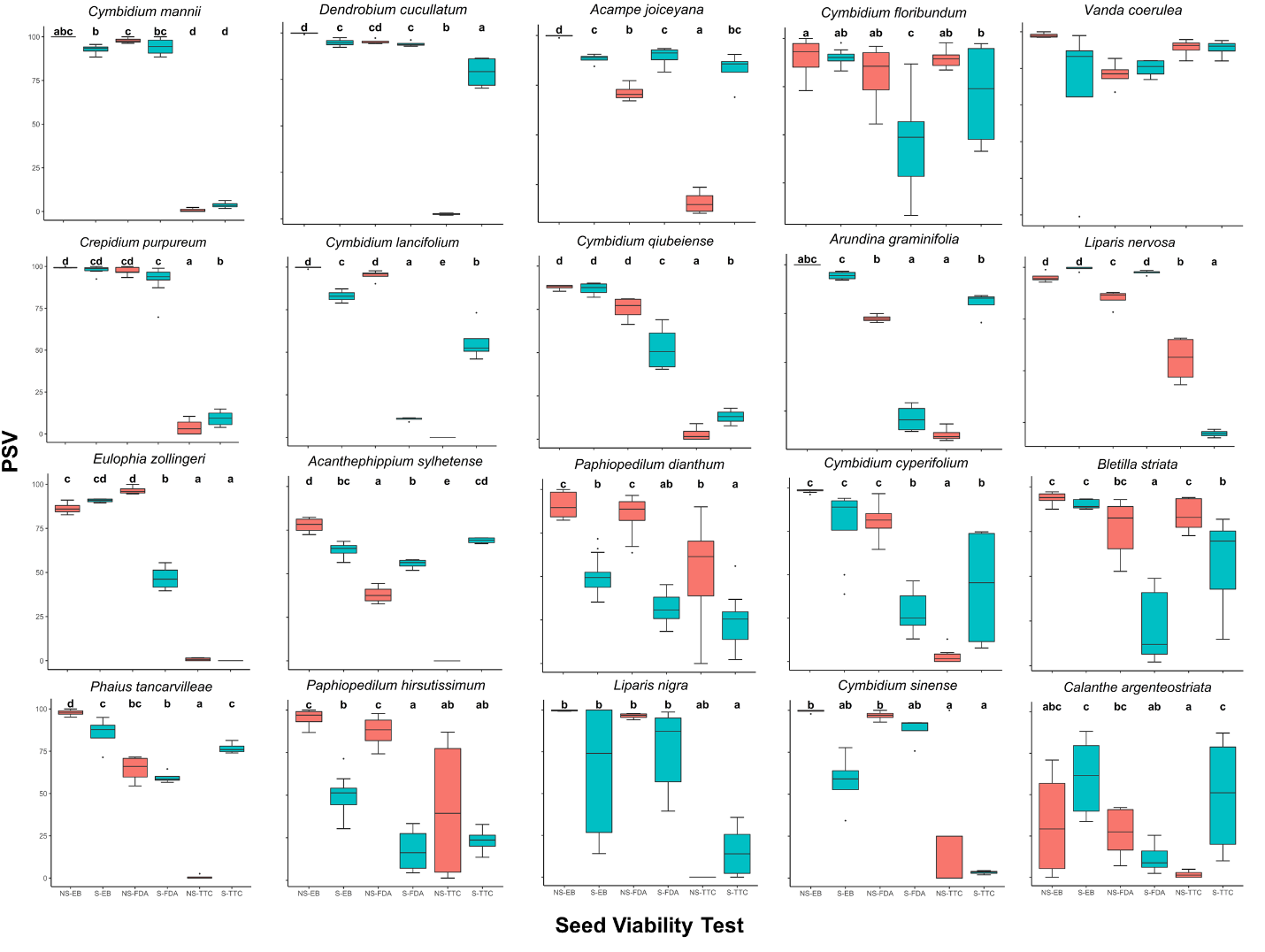


**Figure S1.** Box plot for the probability of finding viable seeds (PSV) after subjecting seeds of five epiphytic (first row) and 15 terrestrial orchid species (second to last row) to three seed viability tests; Evans blue test (EB), Fluorescein diacetate test (FDA) and Tetrazolium test (TTC) without sterilization (NS) and with sterilization (S) of seeds. Viability tests marked with the same letters were similar statistically (*P* > 0.05) while those with different letters were not.
